# Satisfaction with quality of healthcare at primary healthcare settings: Perspectives of patients with type 2 diabetes mellitus

**DOI:** 10.4102/sajp.v76i1.1321

**Published:** 2020-03-05

**Authors:** Tania Steyl

**Affiliations:** 1 Department of Physiotherapy, University of the Western Cape, Cape Town, South Africa

**Keywords:** quality of care, patient satisfaction, type 2 diabetes, primary healthcare, Western Cape, South Africa

## Abstract

**Background:**

Measuring client satisfaction is part of the quality assurance process and has become an integral part of healthcare management strategies globally. It is essential for improvement of amenities in healthcare facilities.

**Objectives:**

The aim of this study was to assess patients with type 2 diabetes’ satisfaction with healthcare services at primary healthcare settings in the Cape Metropolitan district, Western Cape, South Africa.

**Methods:**

This cross-sectional study used proportionate stratified random sampling. The Patient Survey for Quality of Care scale was used to assess patients with type 2 diabetes’ satisfaction with quality of care received. Descriptive and inferential statistics were employed in the analysis of the quantitative data. The open-ended question was analysed qualitatively.

**Results:**

The majority of patients were satisfied with the overall quality of care. Dissatisfaction was reported for waiting time to get appointments at the clinic, to see a healthcare professional on the same day and clarity of instructions for managing their diabetes.

**Conclusion:**

Employing more staff in the multidisciplinary team and improving health information by developing staff through continuous education could foster more positive experiences and provide care that contributes to the well-being of patients.

**Clinical implications:**

Addressing patients’ views regarding quality of healthcare services could assist in overall improvement of healthcare provision through the rectification of the system weaknesses. Satisfaction with quality of care could positively affect adherence to treatment protocols that could lead to better quality of life of patients with type 2 diabetes.

## Introduction

Patient satisfaction is an important aspect of assessing the quality of patient care received at healthcare facilities globally (Al-Abri & Al-Balushi 2014; Phaswana-Mafuya, Davids & Senekal 2011; Sarkar & Chatterjee 2011).

Patient perspectives about the level of care can result in feedback useful for promoting higher quality standards of patient care (Anhang et al. 2014). In Donabedian’s (1988) quality measurement model, patient satisfaction is defined as a patient-reported outcome measure, while the structures and processes of care can be measured by patient-reported experiences (Oyvind, Ingeborg & Hilde 2011). Measuring patient satisfaction is needed to ensure that health services address their needs (Fekadu, Andualem & Yohannes 2011; Mosadeghrad 2014). The process is essential for the improvement of amenities in healthcare facilities (Al-Abri & Al-Balushi 2014; Goldman, Vaiana & Romley 2010).

The classic Quality of Care Framework consists of three elements: structures, process and health outcomes; the latter includes patients’ satisfaction with healthcare services (World Health Organization [WHO] 2000).

The 2012 Institute of Medicine’s landmark report, *Crossing the Quality Chasm*, expands the concept of the Quality of Care Framework to include six aims, namely, patient safety, effectiveness, patient centeredness, timelines, efficiency and equity (Peabody et al. 2006). A key element in the effectiveness of healthcare is the partnership between patients and healthcare providers (De Belvis et al. 2009; McGill & Felton 2007; Nam et al. 2011). Measuring patients’ satisfaction with services supports Donabedian’s (1988) view of the patient as the ultimate authority. The patients’ perceptions of their satisfaction with services provide information on whether the healthcare provider met their expectations (Donabedian 1988). Health-seeking behaviour and adherence to treatment decreases mortality, disability and healthcare costs, and are affected by patients’ satisfaction with services (Shi 2012; Westaway et al. 2003). Valid and reliable health process measurement tools are available, but are not often utilised because of time constraints and inadequate staff numbers (Peabody et al. 2006).

The quality of healthcare services in most developing countries is often poor because of deficiencies in finance and resources (WHO 2000). Most healthcare facilities in sub-Saharan Africa lack healthcare professionals with specialised training in diabetes management, thus potentially compromising the quality of care for patients with this disease (Pastakia et al. 2017). Although a primary healthcare (PHC) approach was integrated into South Africa’s *National Health Act*, 2004 (No. 61 of 2003), and operationalised through the district health service, the prevalence of diabetes mellitus is increasing at an alarming rate. Data from the World Health Organization indicate that 9.8% of South Africans aged 30 years and older have diabetes (WHO 2016). It is important to note that in 2010, the prevalence of type 2 diabetes in South Africa was estimated at 4.5 % (IDF 2009). Thus, an alarming increase is reported in 6 years. Additionally, the reality of the situation is unknown as undiagnosed diabetes affects 84.4% of South Africans aged 20–79 years (IDF 2017). Not all PHC facilities in South Africa have specialised or formally organised diabetes healthcare delivery systems and inefficiently trained staff often manage patients with diabetes, thus compromising the quality of care of patients (Osei 2003).

Patient satisfaction is a key marker of communication and health-related behaviour (Tonio, Joerg & Joachim 2011). Monitoring patients’ perception is an important strategy to improve the performance of healthcare professionals.

Dissatisfaction with healthcare is associated with non-compliance of the treatment regimen and discontinuation of care (Krot & Sousa 2017; Rama & Kanagaluru 2011). Non-adherence, a characteristic of poor patient self-management, can increase mortality and disability, as well as healthcare costs (Currie et al. 2013). The process of providing care in developing countries is often poor and varies widely. Therefore, measuring client satisfaction should be at the forefront of all healthcare facilities.

The overall aim of my study was to assess patients with type 2 diabetes’ satisfaction with healthcare services at PHC settings in the Cape Metropolitan district of the Western Cape, South Africa.

## Method

This cross-sectional study was conducted on patients with type 2 diabetes from PHC facilities in the Cape Metropolitan district of the Western Cape, South Africa.

The study population consisted of all the patients diagnosed with type 2 diabetes mellitus at the community health centres (CHCs) in the Cape Metropolitan district at the time of data collection. Stratified random sampling was employed, the strata being the four sub-structures of the Cape Metropolitan district. To ensure equal representation from each sub-structure, random selection of CHCs was performed proportionately. One CHC was randomly selected from the following three sub-structures: Eastern and Khayelitsha sub-districts, Klipfontein and Mitchells Plain sub-districts, Northern and Tygerberg sub-districts and four CHCs from the Southern and Western sub-districts.

A total of seven CHCs were selected and included in the final sample. The Western Cape Department of Health, however, only gave ethical clearance and permission to conduct the study at six of the selected CHCs. The excluded CHC was inundated with research projects.

The sample size calculation was based on an estimated population, and at 95% confidence level; thus, 378 patients with type 2 diabetes were needed to participate in the study. At each CHC, every second patient who visited the participating clinic for management of his or her disease was approached to participate in the study. Of the 420 patients who were approached, 384 consented and completed the questionnaire. However, 49 questionnaires were not included in the data analysis as they were incomplete. A response rate of 79.76% (*n* = 335/420) was therefore achieved.

## Instrument

Participants were given a modified self-administered questionnaire, based on existing valid and reliable scales.

The questionnaire consisted of two sections, namely, section 1: socio-demographic information and section 2: the patients’ satisfaction with quality care questions and the quality of care scale (Woodward et al. 2000). The scale consists of 10 items with a five-point Likert scale ranging from *poor* to *excellent* (1 = poor, 2 = fair, 3 = good, 4 = very good and 5 = excellent). Higher scores indicate more satisfaction with quality of care received at the CHC. Three questions dichotomised into 0 = no and 1 = yes regarding the CHC, as well as an open-ended statement for suggestions to improve services at the CHC, were included.

A draft of the questionnaire, with all the sub-sections, was translated into isiXhosa and Afrikaans by independent healthcare professionals fluent in the respective languages. The translated questionnaires were then back-translated into English by a linguist in Afrikaans and isiXhosa, respectively. The questionnaires (English, Afrikaans and isiXhosa versions) were administered to a sub-sample of 15 patients with type 2 diabetes mellitus who were not included in the study to assess the face validity and applicability of all the items for this population, its level of understandability and the time it would take to be completed. A 30-min focus group discussion followed the completion of the questionnaire to test the content validity of the instrument and to see whether it was necessary to rephrase or change any of the questions. Only a few grammatical changes were made. The results indicated that the instrument was relevant to the population and was easily used by the patients. The final questionnaire was sent to experts in the field of management of diabetes and chronic diseases of lifestyle to analyse the items to see if they adequately represented the hypothetical content in the correct proportions. No changes were suggested.

To test reliability, the same questionnaire was administered 2 weeks later to the same sub-sample of 15 patients. Test–retest reliability was measured using a correlation coefficient. According to Nunnally and Bernstein (1994), an Intraclass correlation coefficient (ICC) of above 0.81 is considered almost perfect agreement; between 0.61 and 0.80, it indicates substantial agreement; between 0.41 and 0.60, it indicates moderate agreement; between 0.21 and 0.40, it indicates fair agreement; and below 0.20, it indicates poor agreement. The internal consistency of the English, Afrikaans and isiXhosa versions of the translated questionnaires yielded the following scores: 0.748, 0.833 and 0.790, respectively. All the sub-scales, except for the English and isiXhosa versions of the ‘knowledge of diet’ sub-scale, had substantial to almost perfect agreement, indicating good stability.

## Data collection

Data were collected for a period of 6 months, from January to June 2012. After ethical clearance was obtained from all relevant authorities, written informed consent was sought from the 420 patients who were approached to participate in the study. A total of 384 patients consented, while 36 patients refused to participate in the study. The final sample was 335 adult patients with type 2 diabetes mellitus as 49 questionnaires were excluded because of missing data.

Each participant was invited to go to a private room where the anthropometric measurements and blood pressure were taken. The rest of the questionnaire was then completed in the waiting room of the healthcare facility in the presence of the research assistant.

## Data analysis

Data were captured on a 2010 Word Excel spreadsheet and imported into the Statistical Package for the Social Sciences (SPSS) version 21.0 for analysis. Descriptive statistics were employed to summarise the socio-demographic data. Inferential statistics (cross tabulations) were used to determine the pattern of distribution in the various groups. Significant differences were tested using the Fisher’s exact test of independence. Statistical significance was set at a *p*-value of less than 0.05.

The open-ended questions were analysed qualitatively. Content analysis was performed by extracting meaningful ideas of the participants’ opinions and grouping them into categories. Then the author looked for emerging themes. After the derivation of themes, an independent researcher read through the transcripts and generated themes that were then compared with the themes of the author.

## Ethical considerations

Permission and ethical clearance were obtained from the Senate Research Grants and Study Leave Committee at the University of the Western Cape (UWC) (Reference number 11/4/2). Further permission was obtained from the Western Cape Department of Health (2011/07/14; Reference number RP59/2011) and the facility managers of the participating CHCs. The following guidelines were followed:

The consent forms, information sheets and questionnaires were available in Afrikaans, English and isiXhosa.

Signed, written informed consent was sought from all the participants before their participation in the study.

## Results

A total of 335 patients (119 men [35.5%] and 216 women [64.5%] with a mean age of 58.25 [± 10.58 years]) completed the Patient Survey for Quality of Care scale. The participants were predominantly (48.4%) from a mixed race population group. The majority were 50 years and older (81.2%).

Of the participants, 57% were married or had a domestic partner, while half of the participants’ (50.1%) highest level of education was primary school. Participants were asked about where they live most of the year, to which 55.5% reported that they live in their own home or flat, while 39.7% reported they lived with friends or family. More than one-third (37.7%) of the participants were unemployed, while 33.1% were pensioners.

For the purpose of this study, satisfaction was classified as the sum of good, very good and excellent responses and not satisfied was classified as the sum of poor and fair responses. From the data in Table 1, the majority of respondents (78.5%) were satisfied that clinic staff gave clear and complete explanations of medical information. They felt that the staff were concerned and caring (77%) and that their personal safety and their belongings were assured (81.2%). Most of the participants (89.2%) were satisfied with the level of privacy and the overall quality of care (80.9%) provided by the healthcare facility. However, just over half of the respondents were satisfied with the waiting time to get an appointment (52.5%). More than two-thirds of the respondents were not satisfied with waiting time in the clinic room to see a healthcare professional (65.3%); the instructions that were given by clinic staff to prepare for the next visit (69.6%) as well as clarity of instructions to manage their diabetes at home (65.9%).

**TABLE 1:** Satisfaction with the quality care scores of patients with type 2 diabetes mellitus (*n* = 335)

Statement	Poor		Fair		Good		Very good		Excellent	
	*n*	%	*n*	%	*n*	%	*n*	%	*n*	%
Waiting time to get an appointment	29	8.7	130	38.8	169	50.4	7	2.1	-	-
Waiting time in clinic room to see a health care professional	51	15.2	168	50.1	112	33.4	4	1.2	-	-
Instructions given by clinic staff to prepare you for the visit	77	23.0	156	46.6	98	29.3	4	1.2	-	-
Ease of getting information from clinic staff	18	5.4	110	32.8	194	57.9	13	3.9	-	-
Clear and complete explanations on information given by clinic staff	3	0.9	69	20.6	240	71.6	23	6.9	-	-
Concern and caring by clinic staff	5	1.5	72	21.5	168	50.1	90	26.9	-	-
Safety and security of you and belongings	6	1.8	57	17.0	177	52.8	95	28.4	-	-
Privacy	3	0.9	33	9.9	187	55.8	112	33.4	-	-
Clarity of instructions upon leaving	53	15.8	168	50.1	99	29.6	15	4.5	-	-
Overall quality care	4	1.2	60	17.9	217	64.8	54	16.1	-	-

Fisher’s exact test of independence was conducted to compare the statements or items for satisfaction with quality care for men and women. No significant gender differences were found on any of the satisfaction with quality of care outcomes (*p* > 0.05) (Table 2).

**TABLE 2:** Gender differences in satisfaction with the quality care statements of patients with type 2 diabetes mellitus (*n* = 335)

Statement	Gender		Poor		Fair		Good		Very good		Excellent		*P*-value
	*n*	%	*n*	%	*n*	%	*n*	%	*n*	%	*n*	%	
**Waiting time to get an appointment**													0.672
Male	119	35.5	9	7.6	49	41.2	60	50.4	1	0.8	-	-	-
Female	216	64.5	20	9.3	81	37.5	109	50.5	6	2.7	-	-	-
**Waiting time in clinic room to see a health care professional**													0.055
Male	-	-	12	10.1	69	58.0	38	31.9	-	-	-	-	-
Female	-	-	39	18.1	99	45.7	74	34.3	4	1.9	-	-	-
**Instructions given by clinic staff to prepare you for the visit**													0.983
Male	-	-	27	22.7	57	47.9	34	28.6	1	0.08	-	-	-
Female	-	-	50	23.2	99	45.8	64	29.6	3	1.4	-	-	-
**Ease of getting information from clinic staff**													0.379
Male	-	-	3	2.5	39	32.8	72	60.5	5	4.2	-	-	-
Female	-	-	15	6.9	110	32.9	112	56.5	8	3.7	-	-	-
**Clear and complete explanations on information given by clinic staff**													0.658
Male	-	-	-	-	23	19.3	89	74.8	7	5.9	-	-	-
Female	-	-	3	1.4	46	21.3	151	69.9	16	7.4	-	-	-
**Concern and caring by clinic staff**													0.338
Male	-	-	-	-	24	20.2	59	49.6	36	30.2	-	-	-
Female	-	-	5	2.3	48	22.2	168	50.5	54	25.0	-	-	-
**Safety and security of you and belongings**													0.333
Male	-	-	1	0.8	16	13.4	70	67.2	32	26.6	-	-	-
Female	-	-	5	2.3	41	19.0	107	49.5	63	29.2	-	-	-
**Privacy**													0.302
Male	-	-	-	-	8	6.7	71	56.7	40	33.6	-	-	-
Female	-	-	3	1.4	25	11.6	116	53.7	72	33.3	-	-	-
**Clarity of instructions upon leaving**													0.555
Male	-	-	15	12.6	62	52.1	38	31.9	4	3.4	-	-	-
Female	-	-	38	17.6	106	49.1	61	28.2	11	5.1	-	-	-
**Overall quality care**													0.883
Male	-	-	1	0.8	19	16.0	80	67.2	19	16.2	-	-	-
Female	-	-	3	1.4	41	19.0	137	63.4	35	16.2	-	-	-

Responses to the three validity items indicated that only a few patients felt that they were asked to leave the clinic before they felt ready to do so (*n* = 9, 2.7%). Forty-two respondents (12.5%) stated that they sought medical attention as a result of the services rendered at the CHC, while only five (1.5%) stated they would not recommend the clinic to family and friends if they were in need of such services.

Table 3 summarises the responses to an open-ended question regarding their suggestions about any improvement of services at the CHCs. Only 37 (11.0%) participants responded to this question. The identified themes included staff concerns, concerns of time and communication issues.

**TABLE 3:** Summary to open-ended question: Any suggestions for improvement of services at the CHC (*n* = 37)

Main themes	Categories	*n*	%
Staff concerns	More staff needed/available in general, especially increased availability of doctors	35	94.6
	More staff working in the pharmacy of the CHC	13	35.1
	Doctors spending more time with a patient	22	59.5
	More friendly and helpful nursing/administrative staff	26	70.3
Concerns of time	Shorter waiting period for an appointment	17	46.0
	Shorter waiting time at the CHC in general	21	56.6
	Shorter waiting time at the pharmacy	33	89.6
	Longer business hours	16	43.2
Communication issues	More accurate instructions while at the clinic	11	29.7
	More accurate instructions when leaving	19	51.4
	More feedback and communication from the doctor	31	83.8
	Better telephonic access to information needed	14	37.8

CHC, community health centre

With regard to *staff concerns*, almost all the patients suggested that more staff is needed or should be made available to them in general, especially an increased availability of doctors (*n* = 35, 94.6%). Other staff concerns raised were more staff working in the pharmacy of the CHC (*n* = 13, 35.1%), doctors spending more time with patients (*n* = 22, 59.5%) as well as more friendly and helpful nursing or administrative staff (*n* = 26, 70.3%).

Suggestions for *time concerns* included a shorter waiting period for an appointment (*n* = 17, 46.0%) and waiting time at the CHC (*n* = 21, 56.6%) in general as well as at the pharmacy (*n* = 33, 89.2%). Longer business hours (*n* = 16, 43.2%) were also recommended.

Patients advised that *communication issues* should be addressed as follows: more accurate instructions while at the clinic (*n* = 11, 29.7%) as well as when leaving (*n* = 19, 51.4%), more feedback and communication from the doctor (*n* = 31, 83.8%) as well as better telephonic access to information regarding their health concerns (*n* = 14, 37.8%).

## Discussion

The results of this study should be interpreted with caution as they may tend to exaggerate social desirability which may lead to bias. The difference in satisfaction amongst different studies can be attributed to different settings, study participants (not only patients with chronic illnesses) and methodologies employed. The overall level of satisfaction with the healthcare of the patients attending PHC settings in the Cape Metropolitan district of the Western Cape, South Africa, was 80.9%. This finding is similar to a study conducted in Majmaah, Saudi Arabia, where an 81.7% level of satisfaction with healthcare was reported (Mohamed et al. 2015). This study revealed much higher satisfaction with healthcare compared to findings from studies conducted in the United Kingdon (UK), India, Kosovo, Iraq and Botswana where levels of 61.3%, 66%, 73.5%, 50.9% and 57.1% were reported, respectively (AbdSa’adoon, Hussien & Museher 2008; Abiodun 2013; Raghunath, Vijayalakshmi & Sathagurunath 2013; Tahiri et al. 2014; Wetmore et al. 2014).

More than three quarters (78.5%) of the patients were satisfied with the provision of information about their health problems, which is similar to the findings of Bamidele, Hoque and Van der Heever (2011), Fekadu et al. (2011) as well as a study conducted in Swedish PHC settings (Johansson, Bendtsen & Akerlin 2005). Despite the patients’ high ranking of provision of information, many healthcare professionals still struggle to fully understand patients’ information needs (Wadhwa 2002). This author furthermore stated that if healthcare professionals do not provide the correct information regarding the patient’s disease and its management, it could negatively impact patient’s perception of the quality of care received (Wadhwa 2002). Effective communication and clear explanations have the strongest impact in improving overall patient satisfaction amongst other attributes of care (John, Anne & Austin 2003). This finding provides evidence of the importance of the role of communication as a significant determinant of overall patient satisfaction. Interpersonal communication skills of healthcare professionals in terms of their attitude, explanation of conditions, emotional support and involving patients in decision-making are also found to influence patient satisfaction (Andrabi et al. 2012).

This study reported a high satisfaction rate for privacy (89.2%), which is higher than the 69% of participants who were satisfied with the privacy in hospital (Fekadu et al. 2011). Dissatisfaction with privacy was also reported in studies conducted in India (Kumari et al. 2009) and Bangladesh (Mendoza, Piechulek & Al-Sabir 2001). The higher satisfaction rate of this study could be attributed to the research setting. Patients are seen by the healthcare professionals in a confined private space, while the environment of a tertiary hospital is less conducive to privacy. Most of the rooms in a tertiary hospital are four- or six-bed rooms, with healthcare professionals providing their services at the bedside. All the other patients in the room can thus hear conversations between patients and healthcare professionals.

Waiting time – to get an appointment at the CHC, the time to see a healthcare professional or the time a healthcare professional spends during the visit – was of concern for these participants.

Fifty-three per cent of patients were dissatisfied by the overall waiting time to get an appointment as well as the time spent at the CHC. The percentage dissatisfaction is 63.9% reported in a study conducted in Botswana (Bamidele et al. 2011). Waiting time is a cause of frustration for patients and remains a challenge to the quality of care and services in clinics (McCarthy, McGee & O’Boyle 2004). Although only 37.0% of the participants in the Ethiopian study of Fekadu et al. (2011) reported dissatisfaction with waiting time, the authors noted that the ‘time factor’ (time spent on scheduling and waiting time in the clinic) significantly correlated with overall satisfaction.

Satisfaction was also inversely related with waiting time in studies from India (Kumari et al. 2009) and Bangladesh (Mendoza et al. 2001). The major dissatisfaction in out-patient departments at tertiary hospitals is the long waiting time and overcrowded registration (Arshad et al. 2012; Bakshi 2013).

Eighty-seven per cent of patients in PHC settings in Kuwait reported that the communication time between doctors and patients is insufficient (Alhashem, Alquraini & Chowdhury 2011). If enough time is spent with the doctor and the patient’s questions and concerns are addressed, it can lead to a greater belief that the diagnosis and treatment must be correct (Otani, Kurz & Harris 2005). This aspect of care should thus receive careful attention in efforts to improve patient satisfaction. In addition, the dissatisfaction rate with time in this study could be because of the low number of healthcare professionals working at the CHC, hence the higher healthcare professional or patient ratio and patient load.

No significant associations were found between socio-demographic characteristics such as educational status, age, gender and patients’ perceptions of the quality of diabetes care. However, male participants were more satisfied than female participants with the overall quality of care. In a Turkish study, income level (high), marital status (married) and occupation (high level of education) were found to be significant predictors of satisfaction of care (Baltaci et al. 2013). However, Mohamed et al. (2015) and Fekadu et al. (2011) report that patients who are illiterate or have a low level of education and those from an older age group are more satisfied compared to those who have tertiary education and who are from a younger age group, respectively.

### Practical implications

A better appreciation of the factors pertaining to patient satisfaction with quality of healthcare could result in implementation of plans more appropriate for the requirements of patients. Patients are the best arbiters of services because they accurately assess the services provided and their input can help in the overall improvement of healthcare provision. These study results are important considerations for the management of healthcare services to develop policies and strategies for improving the quality of healthcare.

### Limitations of the study

Data were based on self-report; thus, the findings are subject to desirability and recall bias. Although the baseline data of the study were collected from six randomly selected CHCs in the Cape Metropolitan district of the Western Cape, only 37 of the 335 participants completed the open-ended questions. The responses may not be representative of the general population of individuals with type 2 diabetes mellitus. Therefore, generalisation of the findings to other areas is limited.

## Conclusion

The results of this study showed that participants were satisfied with the quality of care provided by the different service providers of the healthcare facility in the Western Cape. There is a need for interventions in terms of time spent at the facility, staff shortage and telephonic access to health outcomes. Such interventions could promote good client-centred service delivery, which could aid in preserving and promoting the well-being and quality of life of patients with type 2 diabetes. The Department of Health’s policy on quality in healthcare (Department of Health 2007) and the South African 2014/2015 Health Review reiterate that public services need to respond to patients’ requests and expectations. It is important that the relevant authorities address the shortages of staff at PHC facilities in the Western Cape. The feedback from patient satisfaction surveys could assist with improved prioritisation and allocation of resources and it could also serve as a platform for providing better services.
